# Stories behind drug discovery: Key figures, cutting-edge technologies, and blockbuster drugs

**DOI:** 10.1016/j.xinn.2025.101018

**Published:** 2025-07-03

**Authors:** Bingjie Li, Yequn Chen, Linlin Shi, Erik De Clercq, Peng Zhan, Bin Yu

**Affiliations:** 1 Department of Cardiology, First Affiliated Hospital of Shantou University Medical College, Shantou 515041, China; 2 State Key Laboratory of Metabolic Dysregulation & Prevention and Treatment of Esophageal Cancer, State Key Laboratory of Antiviral Drugs, Tianjian Laboratory of Advanced Biomedical Sciences, Pingyuan Laboratory, College of Chemistry, Zhengzhou University, Zhengzhou 450001, China; 3 Key Laboratory of Chemical Biology (Ministry of Education), Department of Medicinal Chemistry, School of Pharmaceutical Sciences, Cheeloo College of Medicine, Shandong University, Jinan 250012, China; 4 Laboratory of Virology and Chemotherapy, Rega Institute for Medical Research, 3000 Leuven, Belgium

## Main text

**Figure 1:**
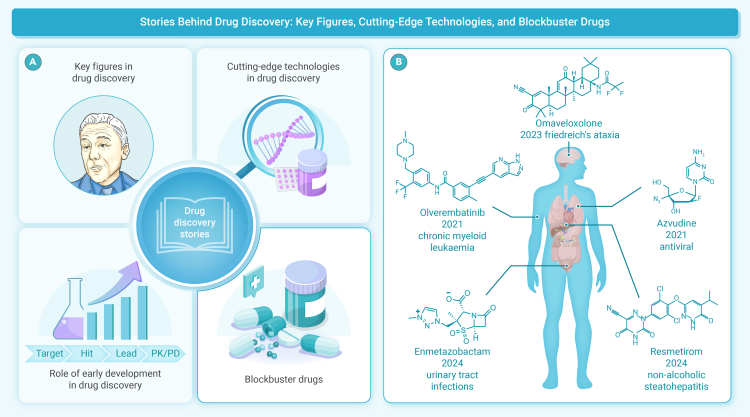
Drug discovery stories (A) Four key contents included in this volume such as key figures, cutting-edge technologies, the roles of early development in successful drug discovery, and blockbuster drugs. (B) Representative drugs approved against various diseases.

The field of drug discovery and development has witnessed remarkable achievements over the past few decades.^1^ The advent of artificial intelligence (AI) is revolutionizing drug discovery by reducing costs and enhancing efficiency.^2^^,^^3^ Nevertheless, drug discovery remains a challenging process with high failure rates, which makes these innovations critical for success. “Drug Discovery Stories (Volume 2),” edited together by Prof. Bin Yu from Zhengzhou University and Prof. Peng Zhan from Shandong University, provides a comprehensive collection of key breakthroughs in contemporary drug discovery (47 chapters).^4^ Following the publication of volume 1 in 2023,^5^ volume 2 mainly includes four fundamental aspects (Figure 1): (1) key figures who have shaped the pharmaceutical industry, (2) the critical roles of early development in successful drug discovery, (3) cutting-edge technologies, and (4) blockbuster drugs approved and selected candidates at the clinical stage. By exploring these themes, this volume aims to commemorate key individuals and highlight recent advances in drug discovery.

## Key figures in drug discovery

Key figures in drug discovery have been essential in shaping the pharmaceutical industry. In chapter 1, Prof. Erik De Clercq recalled his career life with Prof. Piet De Somer, who played a pivotal role in the production of penicillin in Belgium. He also helped make polio vaccines by implementing the methodologies of Jonas Salk and Albert Sabin. His work at KU Leuven, including the establishment of the Rega Institute for Medical Research, was instrumental in strengthening Belgium’s pharmaceutical research landscape. Furthermore, Prof. De Somer was an early advocate for interferon-based therapies, championing interferon as a potential antiviral tool and contributing to research that paved the way for modern antiviral developments. Beyond his scientific discoveries, Prof. De Somer fostered a culture of innovation by mentoring future generations of virologists and medicinal chemists.

## The role of early development in drug discovery success

Early-stage development serves as the foundation that translates laboratory discoveries into viable therapies. This phase involves hit-to-lead optimization, pharmacokinetics (PK)/pharmacodynamics (PD) studies, safety assessments, lead candidate selection, etc. By systematically addressing potential risks and therapeutic benefits early on, drug hunters can identify problematic candidates sooner and mitigate costly late-stage failures. In chapter 3, Dr. Eckhard von Keutz, former Senior Vice President and Head of Translational Sciences at Bayer AG, stated that new technologies, computational sciences, and evolving regulatory requirements are emerging. These developments have the potential to significantly impact early predictions and reduce attrition rates in drug development.

## AI technologies in drug discovery

Applications of AI span multiple stages of drug research and development (R&D). Early successes of AI underscore its potential to significantly shorten the drug development timeline. In chapter 2, Prof. Antonio Lavecchia from University of Naples Federico II highlighted AI’s transformative potential in driving pharmaceutical innovation. He also delved into AI’s pivotal roles at key stages of drug R&D, including target identification, drug design, hit-to-lead optimization, and preclinical studies. Additionally, he discussed challenges in fully adopting AI, such as data quality issues, model transparency concerns, and integrating it effectively into current workflows, and suggested strategies to overcome these obstacles.

## Blockbuster drugs by clinical indications

### Oncology

Modern oncology drug discovery often focuses on designing targeted therapies for novel therapeutics. Representative examples include olverembatinib, repotrectinib, fruquintinib, belzutifan, fluzoparib, JPH-203, STX-478, RLY-2608, LOXO-783, and RNK05047. Hematologic diseases have also benefited from innovative therapies, such as cell-based treatments. Noteworthy examples are idecabtagene vicleucel, momelotinib, imetelstat, and zanubrutinib.

### Infectious diseases

The rapid development of antivirals for infectious diseases such as COVID-19 exemplifies how modern techniques can yield life-saving drugs. In chapter 5, Prof. Erik De Clercq provided a historical perspective on the conception of antivirals and summarized the antiviral drugs that he contributed to bringing from bench to bedside. Other antiviral drugs or candidates include azvudine, ensitrelvir, leritrelvir, remdesivir, cabotegravir, bulevirtide, and mosnodenvir. The rise of antimicrobial resistance is spurring the development of new antibiotics to treat drug-resistant infections. Researchers are developing new β*-*lactamase inhibitors for resistant tuberculosis. Recent advances include enmetazobactam, taniborbactam, rezafungin, etc.

### Metabolic disorders

The drugs for metabolic disorders like type 2 diabetes and obesity have seen breakthroughs. The advent of glucagon-like peptide-1 (GLP-1) receptor agonists has revolutionized diabetes treatment. Alongside GLP-1 therapies, novel insulin sensitizers and oral agents are expanding the toolkits for metabolic disease. Notable examples include semaglutide, chiglitazar, and danuglipron. Another breakthrough is the approval of resmetirom for non-alcoholic steatohepatitis (NASH). As a first-in-class thyroid hormone receptor-β (THR-β) agonist, resmetirom works by enhancing liver metabolism and is the first therapy to directly address the disease process in NASH. Its approval also validates THR-β as a therapeutic target in metabolic liver disease.

### Neurological diseases

Researchers are exploring new ways to enhance cognitive function in patients with neurodegenerative diseases like Alzheimer’s and rare genetic disorders. Phosphodiesterase-4 (PDE4) inhibitors are being investigated for memory enhancement in Alzheimer’s disease. Meanwhile, breakthrough therapies for rare neurodegenerative conditions are emerging. Key examples include omaveloxolone, roflumilast, and donepezil. Another notable example is gepirone, a selective oral 5-hydroxytryptamine receptor 1A (5-HT1A) partial agonist for the treatment of depression. Unlike typical antidepressants that prevent neurotransmitter reuptake, gepirone directly modulates serotonin signaling by partially stimulating 5-HT1A receptors. It represents a potential new class of antidepressants for major depressive disorder. Fingolimod, a sphingosine-1-phosphate (S1P) receptor modulator, represents the first approved oral therapy for relapsing multiple sclerosis (MS). Fingolimod works by sequestering lymphocytes in lymph nodes, preventing them from attacking the central nervous system. Its approval is a breakthrough for patients with MS, offering a convenient oral option and effectively reducing relapse rates and magnetic resonance imaging (MRI) lesions.

### Other diseases

Parasitic diseases have seen significant breakthroughs that improve treatment for affected populations. An important drug is fexinidazole, which is effective against both the first and second stages of the disease caused by *Trypanosoma brucei* and the first all-oral treatment for human African trypanosomiasis. This drug obviates the need for earlier toxic and intravenous therapies, representing a major advancement for a neglected tropical disease. Traditional therapy for chronic kidney disease (CKD)-related anemia involves injectable erythropoiesis-stimulating agents, but new oral drugs that stimulate red blood cell production have been developed. Additionally, rare diseases that affect the blood and kidneys, such as paroxysmal nocturnal hemoglobinuria (PNH), are seeing novel treatments. Key examples include iptacopan and daprodustat. Fezolinetant is the first US Food and Drug Administration (FDA)-approved neurokinin-3 (NK3) receptor antagonist for treating moderate-to-severe vasomotor symptoms associated with menopause. Fezolinetant provides a non-hormonal option for menopause symptom relief. Nerandomilast is an oral, preferential PDE4B inhibitor under development for idiopathic pulmonary fibrosis (IPF). By inhibiting PDE4B, nerandomilast exerts both anti-inflammatory and antifibrotic effects in lung tissue.

## Conclusions and perspectives

“Drug Discovery Stories (Volume 2)”, although not exhaustive, offers a coverage of the scientific breakthroughs that have reshaped the modern pharmaceutical landscape. The contributions of pioneers underscore the importance of visionary leadership in advancing drug discovery. At the same time, AI-driven innovations and other emerging cutting-edge technologies such as proteolysis targeting chimeras (PROTACs) are revolutionizing drug discovery. From the inspiration provided by key historical figures to the sophisticated methods of today, each element plays a role in bringing forth new medicines. The blockbuster drugs discussed showcase the real-world impact of these advancements across diverse therapeutic areas. By leveraging cutting-edge technologies and experiences acquired from past failures and triumphs, researchers are better equipped than ever to innovate new therapies.

## Funding and acknowledgments

We acknowledge financial support from the 10.13039/501100006407Natural Science Foundation of Henan Province (nos. 242301420005 and 252300421243) and the Key Research Project for Basic Research in Henan Province Universities (no. 25ZX001). We are also very grateful to Professor Fener Chen from Fudan University for writing the foreword for this volume and to those who contributed their expertise to this book series.

## Declaration of interests

The authors declare no competing interests.
